# Hot Ductility Prediction Model of Cast Steel with Low-Temperature Transformed Structure during Continuous Casting

**DOI:** 10.3390/ma15103513

**Published:** 2022-05-13

**Authors:** Dae-Geun Hong, Sang-Hum Kwon, Chang-Hee Yim

**Affiliations:** 1 Graduate Institute of Ferrous & Energy Materials Technology, Pohang University of Science and Technology, Pohang 37673, Korea; dghong@postech.ac.kr; 2 Steel Making Research Group, POSCO Research Institute, Pohang 37859, Korea; shkwon@posco.com

**Keywords:** surface crack, bainite start temperature, random forest, machine learning, *N*-shaped fitting

## Abstract

When various alloying elements are added or the cooling rate is increased, steel grades with U- or V-typed ductility behavior show *N*-shaped ductility behavior in which the ductility decreases in the low-temperature region. This study proposes a method that uses *N*-shaped data fitting and random forest to predict ductility behavior of steel grades that have bainite microstructure. To include the phenomenon in which that ductility decreases below the intermediate temperature, the data range was extended to temperature *T* < 700 °C. To identify the *T* range in which the ductility decreases at *T* < 700 °C, an *N*-shaped data fitting method using six parameters was proposed. Comparison with the experimental values confirmed the effectiveness of the proposed model. Also, the model has better ability than models to predict bainite start temperature *T*_BS_. In a case study, the change of ductility behavior according to the cooling rate was observed for Nb-added steel. As the cooling rate increased from 1 °C/s to 10 °C/s, the formation of hard phase was relatively promoted, and different transformation behaviors appeared. This ability to predict the ductility behavior of alloy steels with a bainite microstructure, and to predict *T*_BS_ below the intermediate temperature enables effective control of the secondary cooling conditions during continuous casting process, minimizing the formation of cracks on the slab surface.

## 1. Introduction

During continuous casting, molten steel is poured into a mold that has a certain internal shape; the slab in the mold half-solidifies during passage through the mold, and is continuously extruded from the lower side of the mold to produce semi-finished products of various shapes [1,2]. Hot-charge rolling (HCR) is a variant that can save energy and manpower by charging a high-temperature cast slab from a casting machine directly into a rolling furnace [3,4]. HCR requires cast steel that has no surface defects. However, the operating factors to prevent cracks are not easily controlled, so the cast steel frequently has surface defects [5]. In particular, steels for marine structure, shipbuilding, and pressure vessel are more vulnerable to cracks [6]. This problem requires inspection of the cast slab, and additional processes such as scarfing and grinding, which decrease the productivity of the continuous-casting process and increase the production cost.

Most commercially-produced steel grades have U or V-type ductility curve, as measured using reduction in area (RA) of the cross-section of the gauge section of a specimen at fracture in a tensile test. At the slab cools, ductility is high at temperatures 1100 ≥ *T* ≥ 1000 °C, decreases at 800 ≥ *T* ≥ 700 °C, then increases again at 700 °C > *T*. If *T*_MIN-DUCT_ at which the ductility of steel is lowest can be accurately predicted, surface cracking of the cast steel can be minimized by controlling *T* in the casting machine to be >*T*_MIN-DUCT_ in the bending area and <*T*_MIN-DUCT_ in the unbending area [6]. However, some steel grades that have *N*-type ductility behavior instead of U or V-type, ductility decreases at low temperatures, so the complexity of *T*_S_ control increases.

To minimize the occurrence of cracks on the slab surface, the surface temperature *T*_S_ of the slab must not be allowed to enter the low-ductility range during the bending and unbending processes that occur in the continuous caster [2,5,6,7,8,9]. Tensile tests of each steel can identify the *T* range in which it is brittle (Brittle range). The tests must be performed several times at various *T*, so the time and cost are high [10,11]. Too high cooling rate and alloying amount may reduce cast steel plasticity and lead to crack formation [2,11,12]. Alloying also has an important effect on the ductility of cast steel [10,13]. Even at the same composition, the brittle range can be changed by the thermal history and by the stress applied to the cast steel [7,9,12,13]. Therefore, tests to find the brittle range of the steel must be repeated at different conditions including strain rate and cooling rate. AS a result of these complications, tensile testing of various steel types to identify the brittle range is not practical [6,11,14,15,16,17].

An alternative approach is to develop a model that can predict high-temperature ductility without needing a complicated experimental process. The RA prediction is to minimize cracks of the slab by avoiding the temperature section with low ductility at the bending and unbending stage of the continuous casting process. Methods that have been proposed for this purpose include linear regression [14], multiple linear regression [15], a back-propagation neural network (NN) [16], and deep neural network with a Gaussian curve [6,11]. Existing studies [6,11,14,15,16] have focused on alloy steels in which the ductility behaviors have the general U- or V-shape.

Deep neural network [6,11] collected RA data from web-based academic database and used the Gaussian fitting since more than 70% of the collected data had a U-or V-typed RA pattern. The study converted measured RA values into the low temperature limit (LTL), central temperature (CT) and high temperature limit (HTL) by using only steel grade data with U- or V-shaped ductile behavior from the collected database. The neural network model was selected as the best performance than the other three models such as random forest, gaussian process, support vector machine in all three indicators: LTL, CT, and HTL. However, this prediction model had a limitation in predicting the hot ductility of cast steel with low-temperature transformation structure during continuous casting. When the cooling rate is fast or when various alloy components are added, it is difficult to predict hot ductility with the Gaussian fitting. When a hard phase such as bainite or martensite forms in the cast steel during continuous casting, the ductility of the steel decreases in the low-temperature region [5]. Few studies have tried to predict the ductility behavior of alloy steels in which the ductility has an *N*- or *W*-shape.

So, this paper presents a method to predict the ductility behaviors of steel grades that have an *N*-shaped RA pattern, the method uses the random forest (RF), which is a type of machine learning. The model uses *N*-shaped data fitting with six parameters to identify a *T* below which ductility decreases in the low-temperature region. This study trains and evaluates the RF model using only the steel grade data with *N* and *W* typed ductile behavior from the database of the deep neural network with a Gaussian curve. In the learning process, RF regressor works by building various decision trees and calculating the final average RA value based on different compositions, process conditions, and RA values. To validate performance of the proposed model, representative three machine learning models such as gaussian process, support vector machine, and neural network were selected and compared with RF in terms of prediction accuracy for six parameters. The RF model is also compared with the existing empirical formula to predict bainite start temperature of alloy steel using content of alloying elements. Finally, the effectiveness of the proposed model is verified by comparing RA behavior between the Gaussian model and the *N*-fitting model for the experiment result of Nb-added steel.

## 2. Materials

### 2.1. Data Collection

Data concerned with high-T ductility experiment were gathered from an academic data base such as IEEE Xplore, ScienceDirect, Google Scholar, Springer. The data was comprised of three elements: chemical composition, process variables, and ductility according to *T*. Independent variables have two variables: chemical composition and process condition. The chemical composition consists of 16 variables, and the process condition contains five elements (Table 1). The dependent variable is ductility at 1200 ≥ *T* ≥ 500 °C. Incomplete, duplicate, or unsourced data were eliminated. Finally, 4420 real observed data were obtained (Appendix A).

### 2.2. Data Preprocessing

#### 2.2.1. Data Normalization

In data analysis, if variables have different scales, direct comparison is difficult [18,19], and if they are used in modeling, the differences may distort estimates of their effects. Scale standardization is usually performed to solve these problems. In this study, all for all input and output variables were standardized to range between 0 and 1 by using MinMaxScaler [18]. The initial data of the vector *x* = (*x*_1_, *x*_2_, …, *x_n_*) were standardized as

(1) $$x_{i}^{\prime }=\frac{x_{i}-Min\left(x_{i}\right)}{Max\left(x_{i}\right)-Min\left(x_{i}\right)}$$

where $x_{i}^{\prime }$ stands for the standardized point of *x_i_*, *Max*(*x_i_*) represents the largest point and *Min*(*x_i_*) indicates the least point.

#### 2.2.2. Data Filtering

The ductility data of all of the collected steel grades were visualized using a Python 3.8.4 program language, then 862 data that had *N*-shaped or *W*-shaped ductility curves were selected by inspection of the curves. In addition, deviation of the RA by <10% can occur depending on the experimental equipment or the experimenter, so steel grades that had RA < 10% were excluded. Finally, ductility data vs. *T* of 840 data, i.e., 108 samples (Table 1) were used.

**Figure 1:**
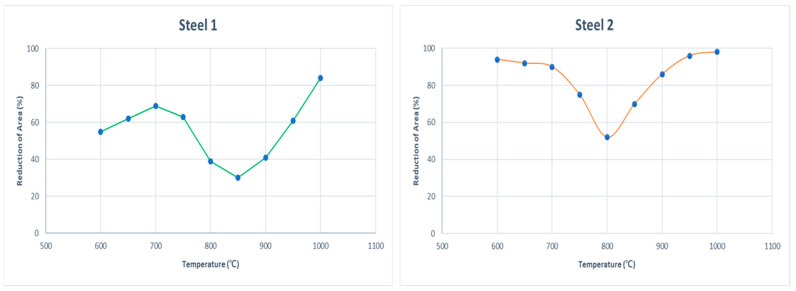
Samples of RA behavior by steel grade.

As an example of data filtering, the ductility behavior for two steel grades was visualized (Figure 1). One was an Nb-added steel with *N*-shaped ductility behavior, and the other was a Nb and Al added steel with U-shaped ductile behavior (Table 2). Both steel grades had the same thermal history. Heating temperature, heat holding time, cooling rate, cool holding time, and strain rate were 1300 °C. 300.0 s, 1.0 °C/s, 0 s, 10^−4^ s, respectively.

## 3. Methods

### 3.1. RF

The random forest algorithm in machine learning is an ensemble technique used for classification and regression analysis, and the basic component of the RF is a decision tree algorithm [20]. The ensemble technique creates one result by collecting several results. The Random Forest maximizes the accuracy of the algorithm by collecting several decision trees and creating a single result [21].

**Figure 2:**
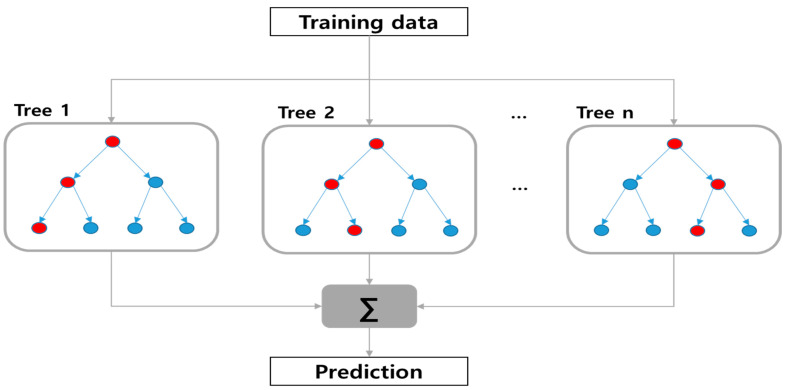
Schematic diagram of RF.

A decision tree has the advantage of being easy to understand intuitively since it is visually expressed as a single tree structure (Figure 2). Only one specific predictor is considered when branches are divided, so the predictive power tends to be low. Also, a small change in the data can lead to a change in the tree structure [21,22]. These traits mean that the bias is relatively low and the variance error is high, so generalization of the model is difficult. Random forest is a machine learning algorithm to compensate for the shortcomings of decision trees.

RF evaluates several decision trees to create a ultimate prediction model. Node t of each tree is classified into a node t_L_ in the left side and a node t_R_ in the right side. The best node division criterion S to extract a desired value is expressed as:

(2) $$S=\mathrm{argmax}\;\Delta î\left(s,\;t\right)=î\left(t\right)-\left[\hat{p}\left(t_{L}\right)î\left(t_{L}\right)+\hat{p}\left(t_{R}\right)î\left(t_{R}\right)\right],$$

where $\hat{p}\left(t\right)$ represents the conditional likelihood at node t, î(t) indicates the impurity function at node t.

(3) $$î\left(t\right)=\frac{1}{n_{t}}\underset{x_{i}\in t}{\sum }{\left(y_{i}-\bar{z_{t}}\right)}^{2},$$

where $n_{t}$ stands for the size of data node t, and $\bar{z_{t}}$ indicates the mean of expected results at node t.

As the impurity difference Δ$\left(î\left(s,\;t\right)\right)$ between child nodes t_R_ and t_L_ increases, the model prediction accuracy increases.

RF iteratively computes independent decision trees by ensuring maximum disorder in sample and variable selection of each model. The bias and variance are reduced as much as possible to reduce the prediction error of the decision tree [21,22]. In addition, interaction and nonlinearity between variables can be considered in high-dimensional data including multiple variables, so RF is stable without causing errors. Therefore, RF improves the accuracy of prediction and also improves generalizability [11,22].

### 3.2. N-Shaped Data Filtering

Data fitting is the task of finding the most appropriate function form with existing data [23,24]. The process can obtain a physical constant from the experimental result, and can determine whether the experimental result or physical model is right or wrong. The problem of fitting data means searching a set *F* of candidate functions to find the function *f* ∈ *F* that is closest to its distribution for *K* data sets as [25,26]:

(4) $$\left(x_{1},\;y_{1}\right),\;\left(x_{2},\;y_{2}\right),\;\cdots ,\;\left(x_{k},\;y_{k}\right)\;\;\left(x_{i}\in D,\;y_{i}\in R\right),$$

where *D* denotes the domain of function *f*, and *R* denotes its range.

The closeness of the function to the distribution of the data set is indicated by the norm of the error *e* between output *y* and the function of input *x* as:

(5) $$e_{p}={\left[\overset{k}{\underset{i-l}{\sum }}{\left|y_{i}-f\left(x_{i}\right)\right|}^{p}\right]}^{\frac{1}{p}},$$

So, the problem of finding the closest function to the given data is to find the function that has the smallest l_p_-norm in Equation (5).

In general, least-squares fitting is a problem of finding a function that has the smallest l_2_-norm, as: [23].

(6) $$minimize\;\overset{k}{\underset{i-1}{\sum }}{\left(y_{i}-f\left(x_{i}\right)\right)}^{2}.$$

**Figure 3:**
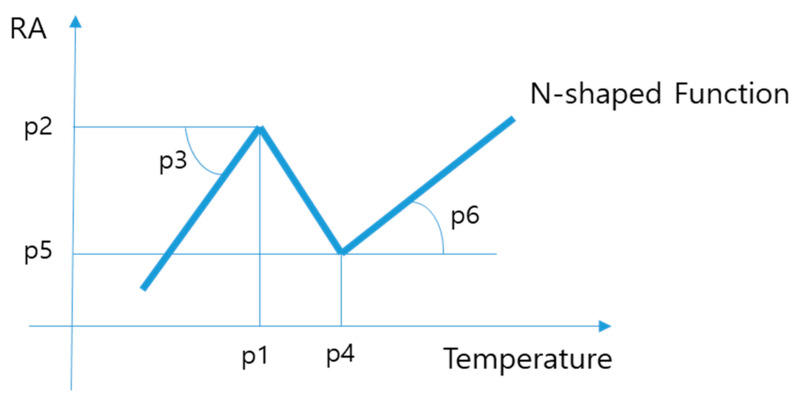
Schematic diagram of functions for fitting *N*-shaped data.

The purpose of this study is to predict the ductility using a RF, and to find a cooling condition that avoids temperature that cause low ductility in the bending/unbending region of a continuous casting machine. For *N*-shaped RA Trough prediction, the collected high-*T* ductility test data were fitted using six parameters (Figure 3): temperature p1 at the low-*T* point, RA p2 at the low-*T* point, slope angle p3 at the low-*T* point, temperature p4 at the high-*T* point), RA p5 at the high-*T* point, and slope p6 angle at the high-*T* point.

**Figure 4:**
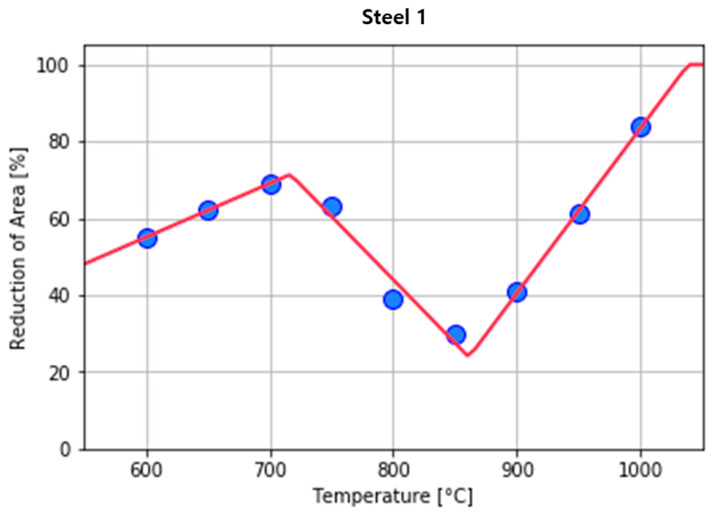
Examples of fitting *N*-shaped data for Steel 1.

Data fitting was performed using the most similar *N*-shape in the RA data collected from individual steel grades. Then the least squares method was used for the six parameters to minimize the sum of squared errors (Figure 4).

### 3.3. Model Optimization and Performance Metrics

Hyper-parameters of all models were adjusted to optimize the proposed model. In the case of RF, the number of decision trees can be adjusted by changing the number of trees (estimators), and the maximum depth of each tree can be optimized by minimizing number of split data [20,27]. In this study, the maximum depth of the tree was set until the output values of the six parameters were obtained, and the minimum number of split data was set to 2 to avoid overfitting. The number of trees was set by trial-and-error method. All models were developed in the Python environment, and RF models were designed using the RFR (Regressor) function of the Scikit-learn library.

RMSE [23,28] was used to quantify the agreement between the observed and predicted values:

(7) $$\mathrm{RMSE}=\sqrt{\frac{1}{n}\overset{n}{\underset{i=1}{\sum }}{\left(x_{i}-y_{i}\right)}^{2}}$$

where *y* stands for the calculated value of the model, *n* is the number of test data, $x_{i}$ indicates the *i*th actual RA value, and $y_{i}$ represents the *i*th predicted value. The prediction accuracy of the model was assessed by applying the RMSE for each of p1, p2, p3, p4, p5, and p6.

## 4. Result and Discussion

### 4.1. Prediction Results Using RF Model

**Figure 5:**
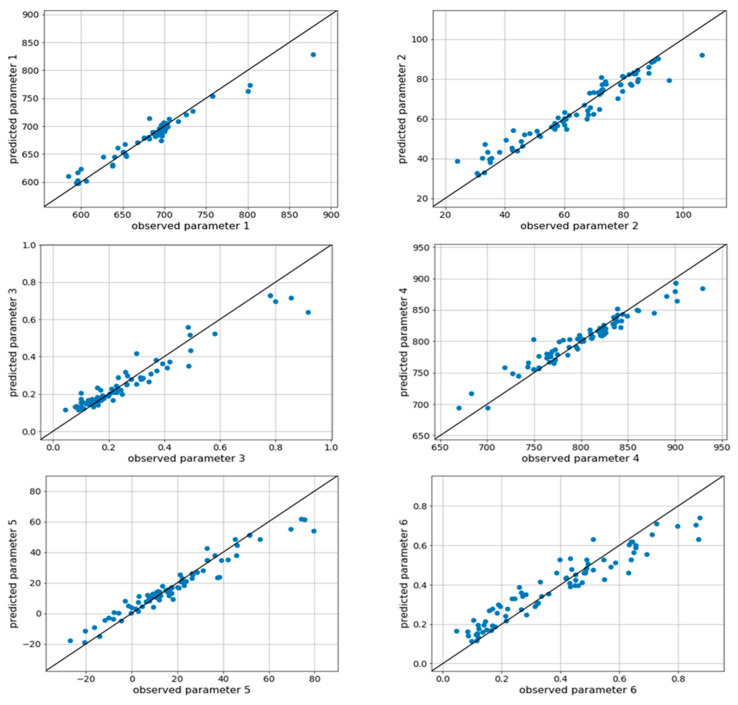
Prediction results for six parameters on test data.

**Figure 6:**
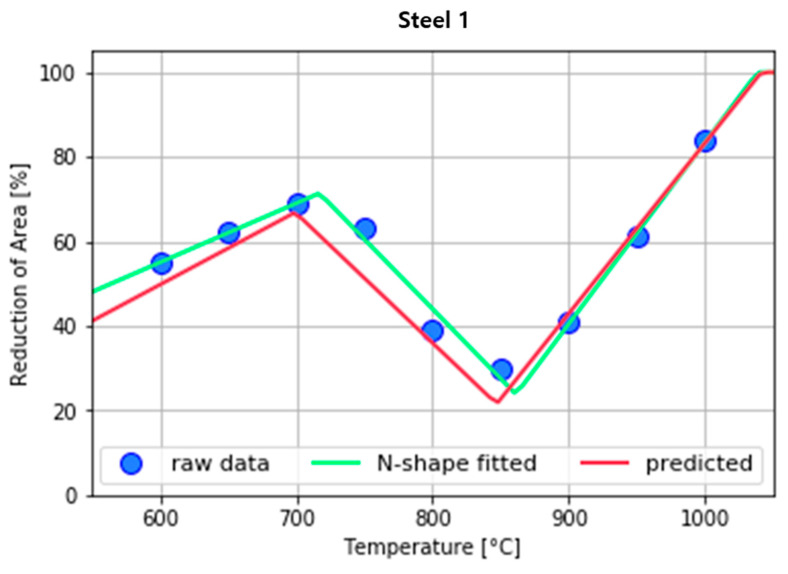
Comparison of RA behavior for Steel 1 using predicted and observed values.

The important hyperparameters of the RF model includes the total size of trees and the maximum height of trees for bagging. As the tree number and depth increase, the model accuracy increases [20,29]. The error of six-fold cross validation was measured while in-creasing the size and height of trees from a low number, and a saturation point was found and set as the optimal point. The number of trees directly affects the computation time and cost in operating the model. The computation time and cost increase as the number of trees increases, so a compromise must be made between model accuracy and the number of trees [29,30]. The average accuracy had a maximum value when the size of trees was 256 and the maximum height was 24. So, the model with 256 trees and a largest height of 24 was selected as optimal. In addition to the two parameters, the error was observed while changing the parameters (min_samples_split and min_samples_leaf). The min_samples_split represents the least number of sample data for dividing a node. The min_samples_leaf is the least number of sample data required to become a leaf node. The changes had no significant effect, so the default values (min_samples_split =2 and min_samples_leaf = 1) were used. The results of the optimized RF model were: p1 RMSE = 11.1, p2 RMSE = 5.11, p3 RMSE = 0.056, p4 RMSE = 15.2, p5 RMSE = 6.11, p6 RMSE = 0.075 (Figure 5). The *N*-shaped RA trough was plotted using the estimates of these six parameters. Then the RA behavior was compared by each steel grade using the observed values in the actual experiment and predicted values of the proposed model (Figure 6). Results confirmed that various types of *N*-shaped RA behavior can be predicted with high accuracy.

### 4.2. Evaluation of Prediction Performance among Four Machine Learning Models

The accuracy of machine-learning models generally depends on the setting of hyper-parameters that control the complexity of the model [11,31]. The hyper-parameter settings must be optimized before the results are compared with those of other models. The k-fold cross validation [32,33] method was applied to optimize the proposed model in this study. The final selected model was evaluated by calculating its accuracy using the test data. The final model for each machine learning model was selected by comparing these results. All analyses used Scikit-learn, a machine-learning library of Python. In this study, four machine-learning algorithms (RF, gaussian process regressor (GPR), support vector regressor machine (SVR), artificial neural network (ANN)) were selected, and their accuracies were compared and analyzed.

First, GPR determines the data relationships between independent and dependent variables by using the mean and covariance function [34,35]. The GP represents the set $f\left(X\right)=\left\{\left(x_{i}),\;\ldots ,\;\left(x_{n}\right)\right\}\right.$ of the surrogate model $f\left(x_{i}\right)$ for the set *X* of the independent variables $x_{i}$. The GPR API supports a combination of several kernels, each of which indicates various properties of the samples. This study evaluated available kernels provided from the API and the WhiteKernel that explains the errors in the samples. The test identified the combination with the Maternkernel showed the best accuracy. Optimal values of hyper-parameters of each kernel were also calculated, and the error level of the Whitekernel was confirmed to be 0.0001. The Maternkernel took the best prediction accuracy when length_scale = 50.0 and nu = 2.5.

Second, support vector machine is a classification algorithm [36] that seeks an optimal hyper-plane that maximizes the margin between categories. SVR used in this study is a generalized technique of support vector machine, and is a technique to predict data by finding the optimal hyperplane between support vectors [37]. SVR used the RBF kernel in this study. Polynomial and sigmoid based kernels were considered, but the RBF kernel de-livered the highest accuracy. The optimal SVR model was calculated by analyzing 10-fold cross-analysis validation while changing C and gamma as a hyper-parameter. The SVR reached the best prediction accuracy when C equaled 2.0 and gamma had a “scale” option. Despite the changes of other hyper-parameters in the kernel, the best accuracy was obtained from the default values.

Third, ANN basically includes an input layer, a hidden layer, and an output layer [38]. ANN has various hyper-parameters such as number of hidden layers, number of nodes in hidden layers, activation function, initializer, learning rate, dropout probability, and batch size [39]. In this study, the model was optimized by changing the number of hidden layers and the number of nodes. The ANN attempted to analyze several hyper-parameters such as number of layers and nodes. In addition, L2 regularization, early stopping, and dropout techniques were applied, but the number of data was too small, so overfitting was difficult to overcome.

**Figure 7:**
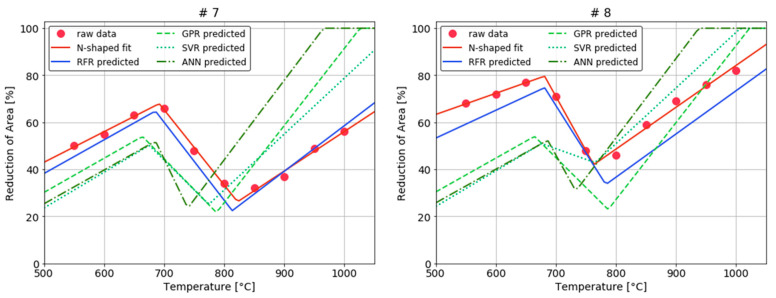
Comparison with performance of four prediction models for *N*-shaped fitting model of steel grade #7 and #8.

The prediction accuracy of four models built on six dependent variables: p1, p2, p3, p4, p5, and p6 was evaluated using RMSE (Table 3). The RF model showed the best pre-diction accuracy for five indicators, and second-best prediction accuracy for P5. The GPR and SVR models had relatively low prediction accuracy compared to the RF model. ANN showed very low prediction accuracy due to overfitting. To check the performance of the four prediction models for the *N*-shaped fitting model, the actual observed RA values, the *N*-shaped fitting model, and the four prediction models were compared for all collected steel grades (Figure 7). In the case of steel grade #7 and #8, the RFR is the closest from the *N*-shaped fitting curve, and the remaining three models are different in distance and behavior.

### 4.3. Prediction of Bainite Start Temperature in Alloy Steel

The bainitic microstructure of steels makes materials that have excellent combinations of mechanical properties including high strength and toughness, creep and fatigue resistance, and hydrogen-embrittlement resistance [40]. The transformation from austenite to bainite begins when the steel reaches the bainite start (Bs) temperature during cooling. This hard phase transformation occurs when the cooling rate is fast, and as an alloying element is added, bainite can be formed even at a low cooling rate. In the continuous casting process, the bainite transformation causes the steel to have an *N*-shaped behavior in which the hot ductility is lowered again in the low-temperature region [5]. Therefore, it is necessary to consider the Bs temperature, which causes the steel to lower again without recovering ductility in the low-temperature region.

Many studies were conducted to analyze the correlation between the addition of alloying elements in steel and the change in Bs temperature. Several researches have been proposed to predict how adding alloying elements to carbon and alloy steels affects the Bs temperature [41,42,43,44,45,46,47,48,49,50]. In this study, six alloying elements C, Mn, Si, Ni, Cr, and Mo were selected for the accuracy comparison with previously-proposed models to predict Bs temperature. The analysis considered only six models that include four or more of the six elements, and no additional components. Four models are linear, one is polynomial, and one is exponential (Table 4). Linear model No. 2 was calculated using the remaining components, excluding Si.

The accuracy of the six existing empirical formulas was compared using data from 108 steel grades that had *N*-shaped or W-shaped ductility vs. temperature. All models had a high error of prediction ≥ 40 °C. Polynomial model No. 5 showed the lowest error (RMSE: 42.9 °C); the remaining models had 47.7 °C ≤ RMSE ≤ 97.6 °C. The large error may occur since the number and distribution of the 862 data used in this study are different from the data sample for which each model was derived.

**Figure 8:**
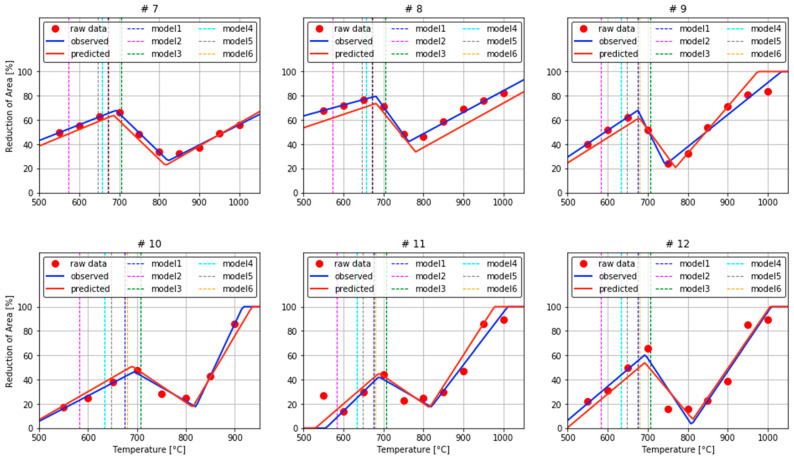
Comparison of the Bs temperature prediction using the existing empirical formula and the *N* shaped fitting model.

The ductility prediction model using *N*-shaped fitting proposed in this study shows more accurate prediction results than the existing Bs temperature prediction models. The estimated Bs temperature closely resembled the measured values (Figure 8). The prediction performance for the temperature of Parameter 1 as the Bs temperature was measured in six empirical formula models and the RFR model, respectively (Table 5). Among the existing empirical models, model5 showed the lowest error, but RFR predicted the Bs temperature more than 30 degrees accurately compared to model5. The more accurately the Bs temperature is predicted, the more effective the secondary cooling conditions can be controlled in the continuous casting machine. This leads to the minimization of cracks in cast steel with low-temperature transformed structure during continuous casting.

However, these results were performed with limited steel-grade data and component elements, so to generalize the model, further studies are needed. Nevertheless, the ductility behavior prediction model proposed in this study was tested with much more steel-grade data than were used to derive the existing empirical formulas. Since the Bs temperature can be changed by the difference not only in composition of the steel but also in cooling rate or austenite grain size, it is difficult to accurately predict the hot ductility only by changing the composition of the steel. Although the range of components is limited to six for comparison with the existing empirical formula, the model proposed in this study can use all 16 components and five thermal histories. Therefore, it can permit prediction of the Bs temperature for a steel grade with a various composition and process condition than the existing empirical formulas can.

### 4.4. Comparison of RA Behavior between Gaussian and N-Fitting Model of Nb-Added Steel

#### 4.4.1. Experimental Procedure

To analyze the phenomenon that steel grades with U/V-shaped RA behavior changes to *N*-typed ductility behavior according to the change of cooling rate, we experimented a steel that had a small amount of Niobium (Table 6), and that was excluded in the collected data base. The most produced steel in the continuous casting process had alloying elements: medium carbon (0.1~0.2%), Si (0.3%), Mn (0.15%), P (0.01%), S (0.05), Nb (0.01%). When an alloy such as Nb was added, the probability of cracking was high. To compare the ductility behavior of U/V- and *N*-shape, one steel grade was selected with experts in the field of continuous casting.

**Figure 9:**
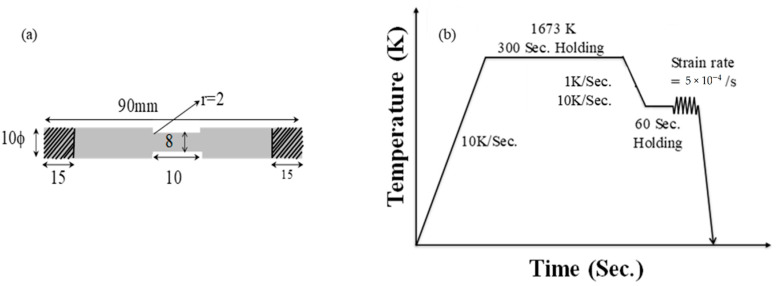
(**a**) Measurement of specimen, and (**b**) condition of tensile experiment.

The test specimens were made from cast steel (Figure 9a). A high-temperature tensile test was conducted by a Caster and Thermo-mechanical simulator (40334, Fuji Electronic Industrial, Saitama, Japan). The first way is to heat up specimens to 1673 K (1400 °C) at 10 K/s to melt precipitates, maintain 300 s at 1673 K, then turn down the heat (873–1273 K) at 1 K/s or 10 K/s (Figure 9b). The second way is to maintain the target temperature for 60 s, then strain rate is applied to the specimen until fracture occurs, and the change in area of two fractured specimen is measured.

#### 4.4.2. Analysis of Result

**Figure 10:**
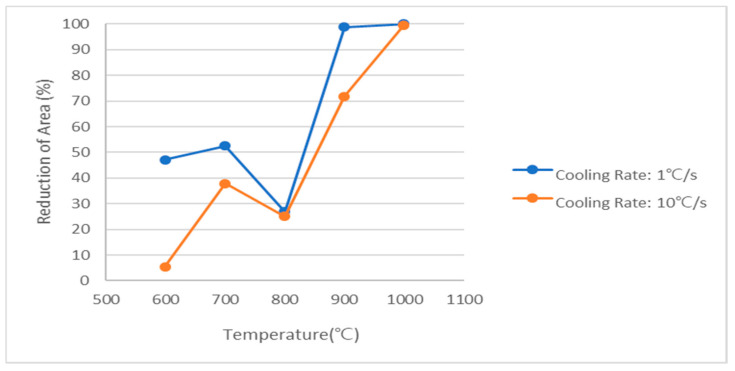
Comparison of RA behavior according to the cooling rate of Nb-added steel.

The tested Nb-added steel has high ductility at high *T* (900 °C–1000 °C) during the cooling process, and exhibits U or V-typed behavior in which ductility is restored again after reaching the lowest value at 800 °C as *T* is lowered. As the cooling rate increased from 1 °C/s to 10 °C/s, the Nb-added steel exhibited a different transformation behavior due to relatively accelerated formation of the hard phase (Figure 10), i.e., the ductility decreased again at 700 °C.

Various alloying elements are added to steel to achieve high strength, weight reduction, and thickening. When the cooling rate of a cast steel having the same composition is increased during continuous casting, a hard phase such as bainite or martensite forms in the steel structure, and the ductility of the steel decreases in the low temperature region [5]. The hard phase is generated at a temperature lower than the general minimum temperature of ductility (700 °C–800 °C), so the decrease in ductility occurs again during the recovery of ductility when the surface of the cast steel is cooled.

**Figure 11:**
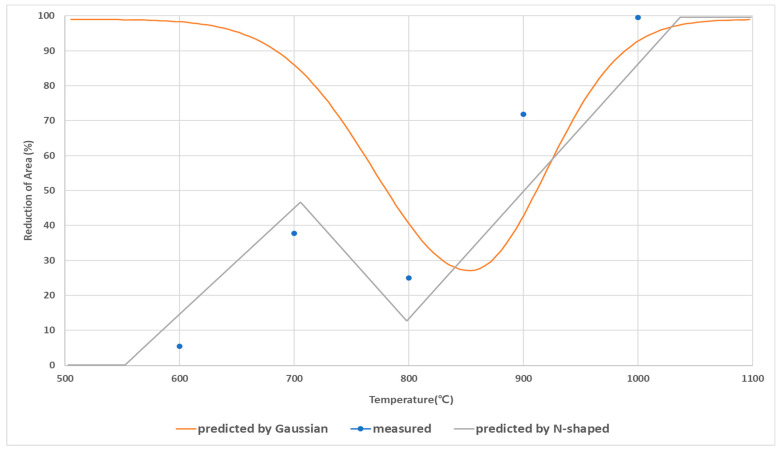
Comparison of RA prediction results of Gaussian and *N*-fitting models and measured RA values.

To check the accuracy of the predicted ductility behavior of Nb-added steel under cooling at 10 °C/s, the RA measured in the experiment were also compared to RA predicts obtained using Gaussian fitting [6] and the *N*-shaped fitting model (Figure 11). Gaussian fitting could not predict the temperature region in which the ductility decreases again at low temperatures (near 700 °C). The RA observed in the experiment at 700 °C and the predicted RA values using the Gaussian function differ by more than a factor of two. In contrast, the RA prediction model using *N*-shaped fitting shows similar the ductility behavior to the observed pattern, and predicted that the ductility would decreases near 700 °C. This result means that Bs temperature can be intuitively checked using the *N*-shaped RA-prediction model.

When various alloying elements are added, or the cooling rate is increased, or the grain size is increased, steel grades with U- or V-typed ductility behavior show *N*-shaped ductility behavior in which the ductility decreases again below a certain temperature. If the temperature at which the ductility of the steel becomes the lowest is accurately predicted [6], the number of surface cracks on cast steel can be minimized by controlling its *T*_S_ higher than that in for the bending part of the casting machine and lower than that in the unbending position. In steels that have *N*-type ductility behavior, precise temperature measurement and control of the slab *T*_S_ are required since the temperature at ductility decreases again is low. The RA-prediction model using the *N*-shaped fitting can help minimize the occurrence of cracks in the slab by increasing precision of the control of *T*_S_ precisely and by indicating the point at which the ductility decreases in the low-temperature region.

For effective temperature control according to the behavior of ductility in the continuous caster, the first task is to identify whether the ductility behavior is U/V-shaped or *N*-shaped. The composition and process conditions of steel lead to changes in the phase transformation temperature and precipitation/segregation behavior as well as the ductility behavior [7,11]. This trait means the hot ductility is a high-dimensional problem between the independent and dependent variables. The ductility of the *N*-shaped steel grade decreases only in a specific temperature range in U- or V-type steel grades, so these types are difficult to distinguish due to the high similarity between the *N*- and the U or V-type curves except in the section where the ductility decreases again (≤700 °C or less).

## 5. Conclusions

This study proposed a method to predict RA for cast steel with low-temperature transformed structure during continuous casting. To simulate the behavior of decreasing RA value in the low-temperature section with the data of composition and process conditions, an *N*-shaped fitting method was proposed and the RA was predicted by random forest, one of machine learning. First, the *N*-shaped RA behavior derived from six parameters and the RA behavior observed in the actual experiments were compared and analyzed. By comparing the predicted values and the observed values for collected steel grades, it was confirmed that the RF model can effectively predict various types of *N*-shaped RA behavior. Second, the prediction performance of *N*-shaped RA behavior was compared using RF, GRP, SVR, and ANN. The difference between the predicted values and the observed values of six parameters for four models was calculated and evaluated by using RMSE. In all other parameters except p5, the predictive performance of the RF model was the best. Third, the Bs temperature was predicted to minimize cracks in cast steel with low-temperature transformed structure during continuous casting. The RF model predicted accurately the Bs temperature more than 30 degrees compared to the empirical formula. The RF model can be a practical alternative to optimally control the secondary cooling conditions of continuous caster. Finally, the change in ductility behavior according to the cooling rate of Nb-added steel was observed. Except for low-carbon steel with low cracking, it is the most produced or widely used carbon region in the continuous casting process. when an alloy such as Nb is added, the probability of cracking is high. The RA prediction model using *N*-shaped fitting not only showed similarly the ductility behavior of Nb-added steel, but also clearly checked that the ductility decreases near 700 °C.

The limitations of this study were also discussed. First, the RF model for predicting *N*-shaped RA curve used the composition and process conditions of 108 steel grades. In the collected RA database, the number of steel grades with *N*-shaped ductility behavior was about 10 to 15%. Due to the limited number of steels, there is some insufficiency in the robustness and adaptability of the RF model. If data are continuously secured, it can be solved by learning and upgrading the RF model. Second, it is not easy to classify the two types of steel grades by using data fitting due to the high similarity between the *N*- and the U/V-typed curves. Data collection is limited by time and cost practically. So, predicting the absolute RA value according to temperature instead of data fitting should be considered in future research.

## Figures and Tables

**Table 1:** Composition [wt.%] and thermal histories of alloys considered in this analysis.

Chemical	Minimum	Maximum	Thermal History	Minimum	Maximum	Unit
Component						
C	0.001	0.52	Heating Temperature	1250	1500	°C
Si	0	0.425	Heat Holding Time	120	300	s
Mn	0	1.9	Cooling Rate	0	10	°C/s
P	0	0.11	Cool Holding Time	0	300	s
S	0	0.015	Strain Rate	0.0001	0.02	1/s
Cu	0	1				
Nb	0	0.078				
Ni	0	1				
Al	0	0.41				
Mo	0	0.093				
N	0	0.016				
Cr	0	1.1				
V	0	0.35				
Ti	0	0.054				
B	0	0.005				
Sn	0	0.192				

**Table 2:** Weight percentage of composition of Nb- and Al-added steel.

Steel		Component					
	C	Si	Mn	P	S	Nb	Al
Steel 1	0.09	0.01	0.01	0.002	-	0.07	-
Steel 2	0.09	0.23	1.40	0.007	0.002	0.02	0.03

**Table 3:** Error range for six parameters calculated from four models.

Model	RMSE					
	p1	p2	p3	p4	p5	p6
RF	11.10	5.11	0.056	15.20	6.11	0.075
GPR	19.22	9.975	0.103	28.01	4.903	0.156
SVR	29.57	10.66	0.132	32.96	12.34	0.156
ANN	71.89	15.95	0.272	58.40	29.97	0.174

**Table 4:** Existing empirical formula to predict bainite start temperature (°C) of alloy steel using content (wt.%) of alloying elements.

No.	Equation	Reference
1	Bs = 830 − 270C − 90Mn − 37Ni − 70Cr − 83Mo	[41]
2	Bs = 656 − 57.7C − 75Si − 35Mn − 15.3Ni − 34Cr − 41.2Mo	[42]
3	Bs = 844 − 597C − 63Mn − 16Ni − 78Cr	[44]
4	Bs = 732 − 202C + 216Si − 85Mn − 37Ni − 47Cr − 39Mo	[46]
5	Bs = 745 − 110C − 59Mn − 39Ni − 68Cr − 106Mo + 17MnNi + 6Cr^2^ + 29Mo^2^	[49]
6	Bs = 839 − 270[1 − exp(−1.33C)] − 86Mn − 23Si − 67Cr − 33Ni − 75Mo	[50]

**Table 5:** Error of six empirical model and RFR for p1.

Bs Temperature	RMSE						
	Model 1	Model 2	Model 3	Model 4	Model 5	Model 6	RFR
P1	51.44	97.60	87.64	55.87	42.90	47.70	11.10

**Table 6:** Weight percentage of composition of Nb-added steel.

Steel	Component						
	C	Si	Mn	P	S	Nb	Cu
Steel	0.16	0.3	0.15	0.01	0.005	0.01	0.03

## Data Availability

The datasets generated during and/or analyzed during the current study are available from the corresponding author on reasonable request.

## Appendix A

**Authors**	**Source**	**Publication** **(Year, Volume, Page)**
Byoungchul Hwang et al.	Materials Science	2005, 402, 177
UnHae Lee et al.	ISIJ International	2010, 50, 540
KyungChul Cho et al.	Metallurgical and Materials Transactions A	2010, 41, 1421
Chihiro Nagasaki and Junji Kihara	ISIJ International	1997, 37, 523
Guoyu Qian et al.	ISIJ International	2014, 54, 1611
Hirowo G. Suzki et al.	ISIJ International	1984, 24, 169
Xia Jiang and Shenhua Song	Materials Science and Engineering A	2014, 613, 171
Jessica Calvo et al.	ISIJ International	2007, 47, 1518
William T. Nactrab and Yu T. Chou	Metallurgical Transactions A	1986, 17, 1995
Israel Mejía et al.	Materials Science and Engineering: A	2011, 528, 4468
Edgar L. Chipres et al.	Materials Science and Engineering: A	2007, 460, 464
Zakaria Mohhamed	Materials Science and Engineering: A	2002, 326, 255
Atef S. Hamada and Pentti Karjalainen	Materials Science and Engineering: A	2011, 528, 1819
Chiaki Ouhi and Kazuaki Matsumoto	ISIJ International	1982, 22, 181
Junru Li et al.	Materials Science and Engineering: A	2016, 677, 274
Thierry Rewaux et al.	ISIJ International	1994, 34, 528
Hang Su et al.	Materials Science and Technology	2013, 23, 1357
Weijian Liu et al.	ISIJ International	2015, 56, 1133
Barrie Mintz	ISIJ International	1999, 39, 833
ShinEon Kang et al.	Materials Science and Technology	2021, 37, 42
Lesley H. Choun and Lesley A. Cornish	Materials Science and Engineering: A	2008, 494, 263
Nowak Wolańska et al.	J. Achievements Mater. Manuf. Eng.	2007, 20, 291
Abdelbaset M. Elwazri et al.	ISIJ International	1999, 39, 253-
Lesley H. Choun	Ph.D. Thesis, University of the Witwatersrand	2008
Kristin R. Capenter	Ph.D. Thesis, University of Wollongong	2004
Haiwen Luo et al.	ISIJ International	2002, 42, 273
Yasuhiro Maehara and Yasuya Ohmori	Materials Science and Engineering	1984, 62, 109
David Crother et al.	Metallurgical Transactions A	1987, 18, 1929
Nils E. Hannerrs	Transactions of the Iron and Steel Institute of Japan	1985
Brendan M. Connolly	Ph.D. Thesis, University of Pittsburgh	2016
Kevin M. Banks et al.	International Journal of Metallurgical Engineering	2013, 2, 188
Kevin M. Banks et al.	Materials Science and Technology	2012, 28, 536
Zuhair Mohjamed	Ph.D. Thesis, City University London	1988
Zuhair Mohjamed	Engineering Journal of University of Qatar	1995, 8, 167
KyungChul Cho	Ph.D. Thesis, Pohang University of Science and Technology	2011
Yaxu Zheng et al.,	Materials Science and Engineering: A	2018, 715, 194
Yutian He et al.	Materials Science and Engineering: A	2016, 673, 99
Xiao-Min Chen et al.	Materials Science and Engineering: A	2010, 527, 2725
Shenhua Song et al.	Journal of Rare Earths	2016, 34, 1062
Shenhua Song et al.	Materials Science and Engineering: A	2003, 360, 96
Jessica Carlvo et al.	Engineering Failure Analysis	2007, 14, 374
Chang-Hoon Lee et al.	Materials Science and Engineering: A	2016, 651, 192
Faramarz Zarandi and Steve Yue	ISIJ International	2006, 46, 591
Xue Jiang et al.	Materials Science and Engineering: A	2013, 574, 46
Shenhua Song et al.	Materials Science and Engineering: A	2014, 595, 188
SangChul Seo et al.	Metals and Materials International	2006, 12, 273
Shin Eon Kang	Ph.D. Thesis, City University London	2014
Jingyu Li and Guoguang Cheng	Journal of Materials Research and Technology	2020, 9, 52
Cheng-bin Shi et al.	Materials Transactions	2016, 57, 647
Eduardo H. Delgado and Rodolfo D. Morales	Metallurgical and Materials Transactions B volume	2001, 32, 919
Ramesses Abusosha et al.	Materials Science and Technology	1999, 15, 278
Monica L. Wang et al.	Kang T’ieh/Iron and Steel	2012
Andrew Cowley et al.	Materials Science and Technology	1998, 14, 1145
Ramesses Abusosha et al.	Materials Science and Technology	1998, 14, 346
Ramesses Abusosha et al.	Materials Science and Technology	1998, 14, 227
Barrie Mintz et al.	Materials Science and Technology	1998, 14, 222
Barrie Mintz et al.	Materials Science and Technology	1995, 11, 474
Barrie Mintz and Ramesses Abushosha	Materials Science and Technology	1992, 8, 171
Ramesses Abusosha et al.	Materials Science and Technology	1991, 7, 613
Chen L. Zhen et al.	Steel Research	1990, 61, 620
Barri Mintz and Mike Arrowsmith	Metals Technology	1979, 6, 24
Chas Spradery and Barrie Mintz	Ironmaking & Steelmaking	2005, 32, 319
Prasonk Sricharoenchai et al.	ISIJ International	1992, 32, 1102
Kyung-Chul Cho et al.	ISIJ International	2010, 50, 839
Barrie Mintz et al.	International Materials Reviews	1991, 36, 187
Barrie Mintz et al.	Materials Science and Technology	2003, 19, 1721
Mustafa M. Arıkan	Metals	2015, 5, 986
Hirowo G. Susuki et al.	ISIJ International	1982, 22, 48
Chunyu He et al.	Metals	2020, 10, 1679
